# Sedimentary logs and facies characterization dataset of Tembeling group from the vicinity area of Kuala Tahan, Pahang, Malaysia

**DOI:** 10.1016/j.dib.2023.109762

**Published:** 2023-11-04

**Authors:** Wei Chung Khor, Kamal Roslam Mohamed, Mohamed Shafeea Leman, Che Aziz Ali

**Affiliations:** a Geology Programme, Faculty of Science and Natural Resources, Universiti Malaysia Sabah, 88400 Kota Kinabalu, Sabah, Malaysia; b Geology Programme, Faculty of Science and Technology, Universiti Kebangsaan Malaysia, 43600 UKM Bangi, Selangor, Malaysia

**Keywords:** Geology, Sedimentology, Facies, Sedimentary logging

## Abstract

This sedimentary logging and facies characterized dataset of 28 outcrops exposed along Kuala Tahan – Kampung Pagi – Kampung Bantal which is situated in the central part of Peninsular Malaysia (in the state of Pahang). This dataset is recorded in 2017 during the construction of roadway. It consist of Mangking Formation of Tembeling Group with the total length of 410 m. The outcrops are arranged into 8 continuous sections. This data can be further correlated stratigraphically to produce composite log, facies analysis, depositional processes, and the depositional environment.

Specifications Table

**Table:** 

Subject	Earth and Planetary Science; Geology; Stratigraphy
Specific subject area	*Geology; Stratigraphy; Sedimentology*
Data format	Processed-Digitized sedimentary logs in Adobe Illustrator^Ⓡ^ (.ai) Raw-Outcrop details in Excel (.xlsx) Raw-Outcrop coordinate (latitude and longitude) Raw-Outcrop photos (.jpg) Processed-Map (.jpg and ArcGis^Ⓡ^)
Type of data	-Table -Figure -Sedimentary logs -Outcrops photos -Map
Data collection	The logs contain length of the outcrops, thickness of beds, observed sedimentary structures and presence of ichnofossils. Orientation of the beddings are recorded as strike and dip. These parameters are digitized as sedimentary log for 28 outcrops (Figures).
Data source location	Country: Malaysia District/Region: Kuala Tahan/Pahang Latitude and longitude: 4.4092900, 102.4354056
Data accessibility	The processed data has been deposited in the Mendeley repository and is accessible using the link: Repository name: Mendeley Data Direct URL to data: https://data.mendeley.com/datasets/3sz9g6nrc8/1

## 1 Value of the Data

• The outcrops and sedimentary logs can inspire researchers to conduct more advanced studies on sedimentology, facies analyses and depositional environment interpretation on Jurassic-Cretaceous rocks of the region.
• The dataset can be stratigraphically correlated with the geology of Peninsula Malaysia, Thailand, and Indochina especially on the Jurassic-Cretaceous aged strata (red beds).
• The outcrops/exposures are now inaccessible due to dense vegetation and cementing through rock/slope stabilization techniques. Other Jurassic-Cretaceous rocks in this region are difficult to access.

## 2 Data Description

In the Mendeley Data repository, there are 4 separate folders, namely, 1) Outcrop Details, 2) Compiled Sedimentary Logs, 3) Locality Map, and 4) Digitization Raw Files. Folder 1 (Outcrop Details) contains excel file with coordinates, bedding orientations (strike and dip) and note on structures (fault and folding). Sedimentary logs and outcrop photos are arranged in Folder 2 (Compiled Sedimentary Logs) based on section (Section 1 to 11) (Figs. 2 and 3). The locality map in Folder 3 (Locality Map) is generated using ArcGIS^Ⓡ^ to show the locations of outcrops (Table 1).

**Table 1:** Summary of the characteristics features of the lithofacies types.

	Facies	Description
Gravelly	Clast-Supported Conglomerate Facies (Gc)	Clast- supported, no imbrication, subangular to subrounded, cobble to coarse pebble sized, erosion surface, gently undulating, lenticular geometries.
	Trough Cross-Stratified Conglomerate Facies (Gt)	Matrix- supported, clasts accumulate along trough cross-bedding cosets, extraformational clasts of mudstone
Sandy	Cross-Stratification Sandstone Facies (St)	Cross-bedding, fine – coarse grained.
	Massive Sandstone Facies (Sm) Subfacies: Smi & Smii	Smi: Thin, structureless, fine to medium grained sandstone. Smii: Thick, crude, structureless, medium to coarse
	Planar Cross-stratified Sandstone Facies (Sp)	Planar stratified, fine to medium grained
Muddy	Parallel Laminated Sandstone Facies (Sh)	Parallel lamination
	Mudstone with Thin Sandstone Stripes Facies (Fl)	Both mudstone- and sandstone- dominated. Sandstones are in stripes shaped
	Mudstone Facies (Fm)	Laminated and massive

(Files and folders shared at Mendeley Data repository: https://data.mendeley.com/datasets/3sz9g6nrc8/1).

The locality of this dataset comprises the Jurassic – Cretaceous-aged rock unit from the Tembeling Group, namely the Mangking Formation. A N-S orientation boundary with Triassic aged Semantan Formation is reported westward. Other Jurassic – Cretaceous geological dataset of Peninsula Malaysia can be obtained from Khor et al. (2017) [1].

The eight sedimentary facies identified are 1) Clast-Supported Conglomerate Facies (Gc), 2) Trough Cross-Stratified Conglomerate (Gt), 3) Trough Cross-Stratified Sandstone Facies (St), 4) Massive Sandstone Facies (Thick: Smi & Thin: Smii), 5) Tabular Cross-Stratified Sandstone Facies (Sp), 6) Parallel Laminated Sandstone (Sh), 7) Interbedded Sandstone and Mudstone Stripes (Fl), and 8) Mudstone Facies (M). Below is a summary of the eight facies (Table 1).

## 3 Experimental Design, Materials and Methods

(i) *Sedimentary logging*

The sedimentary characteristics recorded during field excursion are lithology, grain size, primary sedimentary structures, bedding, external geometry, fossils, thickness, grain size, and color based on Tucker (2011) [3]. Tools used in logging were hand lens, measuring tape, compass, and geological hammer. These details for each outcrop were then digitized using Adobe Illustrator^Ⓡ^ into sedimentary logs (refer Figs. 2 and 3).

(ii) *Facies characterization*

Detailed facies characterization were done using Miall (1996) [2] classification scheme. This classification uses abbreviations such as Sp, Sm, Fl, and Fm and are as listed in the table below (Fig. 4) (Table 2). Beds are classified into these abbreviations based on sedimentary characteristics, notably, bedding, grain size, texture, and sedimentary structures.

(iii) *Locality Map*

**Table 2:** Facies characterization based on Miall (1996).

Facies code	Characteristics	Structure	Interpretation
Gm	Matrix-supported, massive gravel	Non to weak grading	Plastic debris flow (high-strength, viscous)
Gc	Clast-supported gravel	–	Clast-rich debris flow (high strength), or pseudoplastic debris flow (low strength)
Gt	Gravel, stratified	Trough cross-beds	Minor channel fills
Gh	Clast-supported crudely bedded gravel	Horizaontal bedding, imbrication	Longitudinal bedforms, lag deposits,sieve deposits
St	Sand, fine to very coarse, may be pebbly	Solitary or grouped planar cross-beds	Sinous-crested and linguiod (3-D) dunes
Sp	Sand, fine to very coarse, may be pebbly	Solitary or grouped planar cross-beds	Transverse and linguoid bedforms (2-D dunes)
Sm	Sand, fine to coarse	Massive, or faint lamination	Sediment-gravity flow deposits
Sr	Sand, very fine to coarse	Ripple cross-lamination	Ripples (lower flow regime)
Sh	Sand, very fine to coarse, may be pebbly	Horizontal lamination parting or streaming lineation	Plane-bed flow (critical flow)
Sl	Sand, very fine to coarse, may be pebbly	Low angle (<15ᵒ) cross bedding	Scour fills, humpback or washed-out dunes, antidunes
Fl	Sand, silt, mud	Fine lamination, very small ripples	Overbank, abandoned channel, or waning flood deposits
Fm	Mud, silt	Massive, desiccation cracks	Overbank, abandoned channel, or drape deposits

**Fig. 1:**
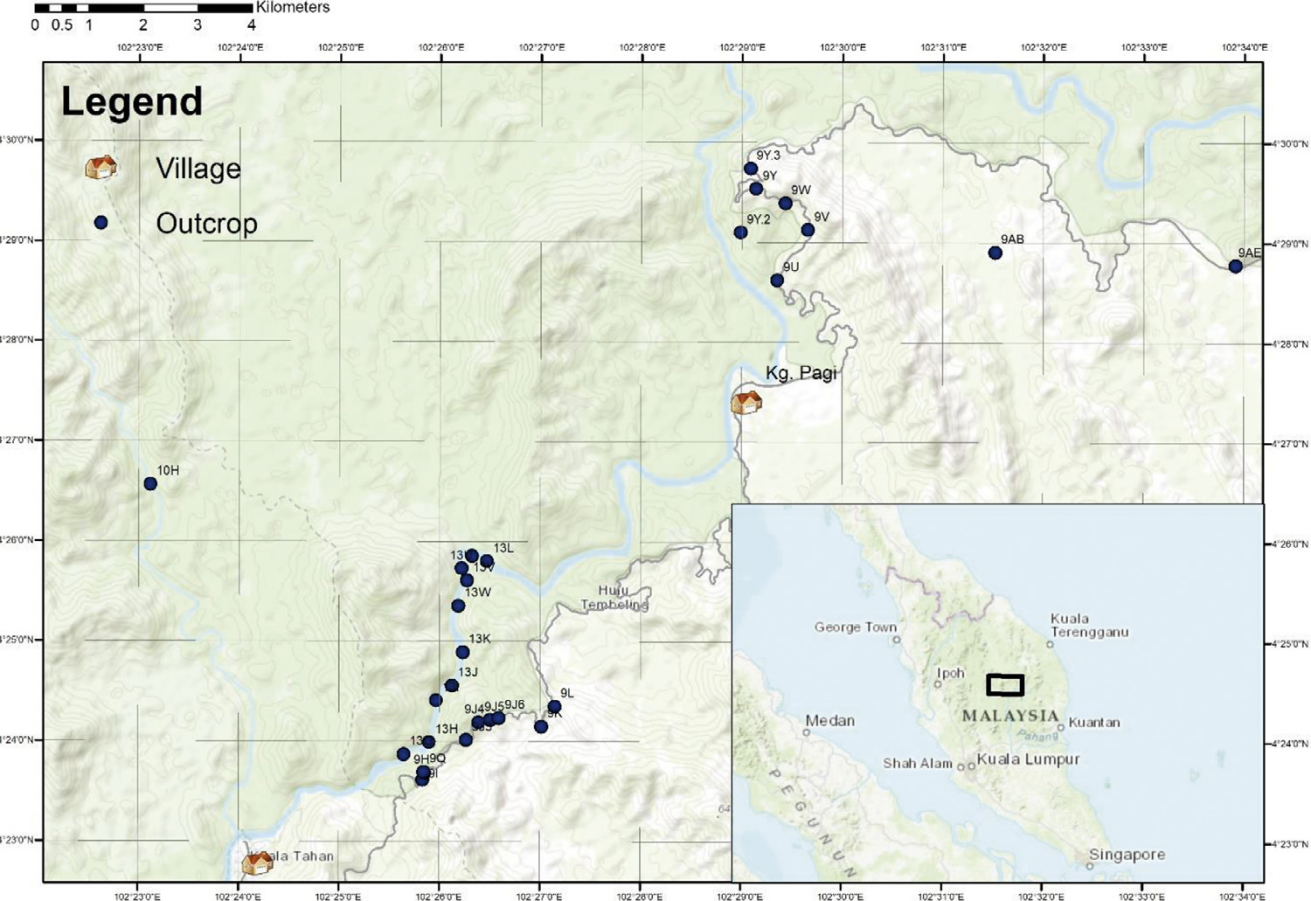
Locality map of outcrops logged in this dataset in ArcGIS^Ⓡ^.

Outcrops were correlated into eight sedimentary sections based on their coordinates, orientation readings, and geological structures.. Coordinate readings of each outcrop were plot using ArcGis^Ⓡ^ (Fig. 1).

**Fig. 2:**
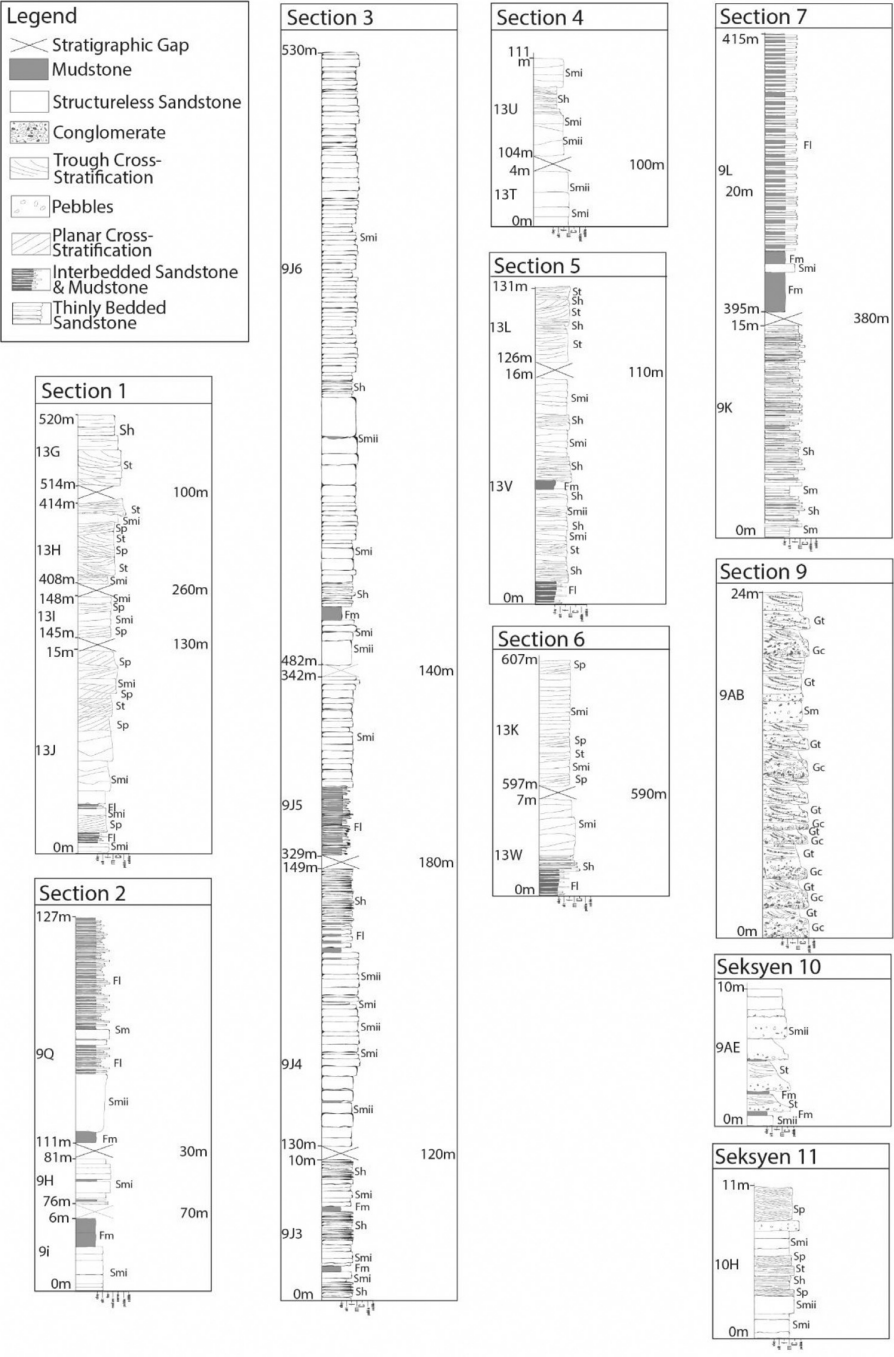
Compilation of sedimentary logs for Section 1, 2, 3, 4, 5, 6, 7, 9, 10 and 11.

**Fig. 3:**
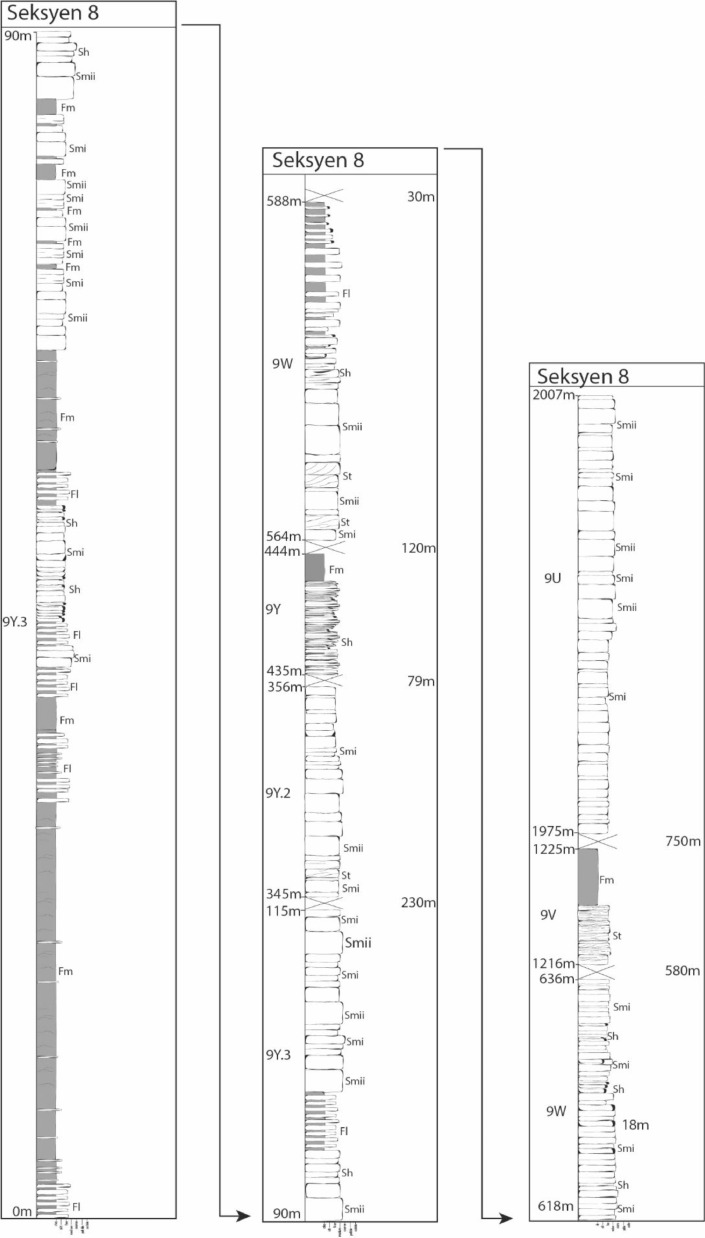
Sedimentary logs for Section 8.

**Fig. 4:**
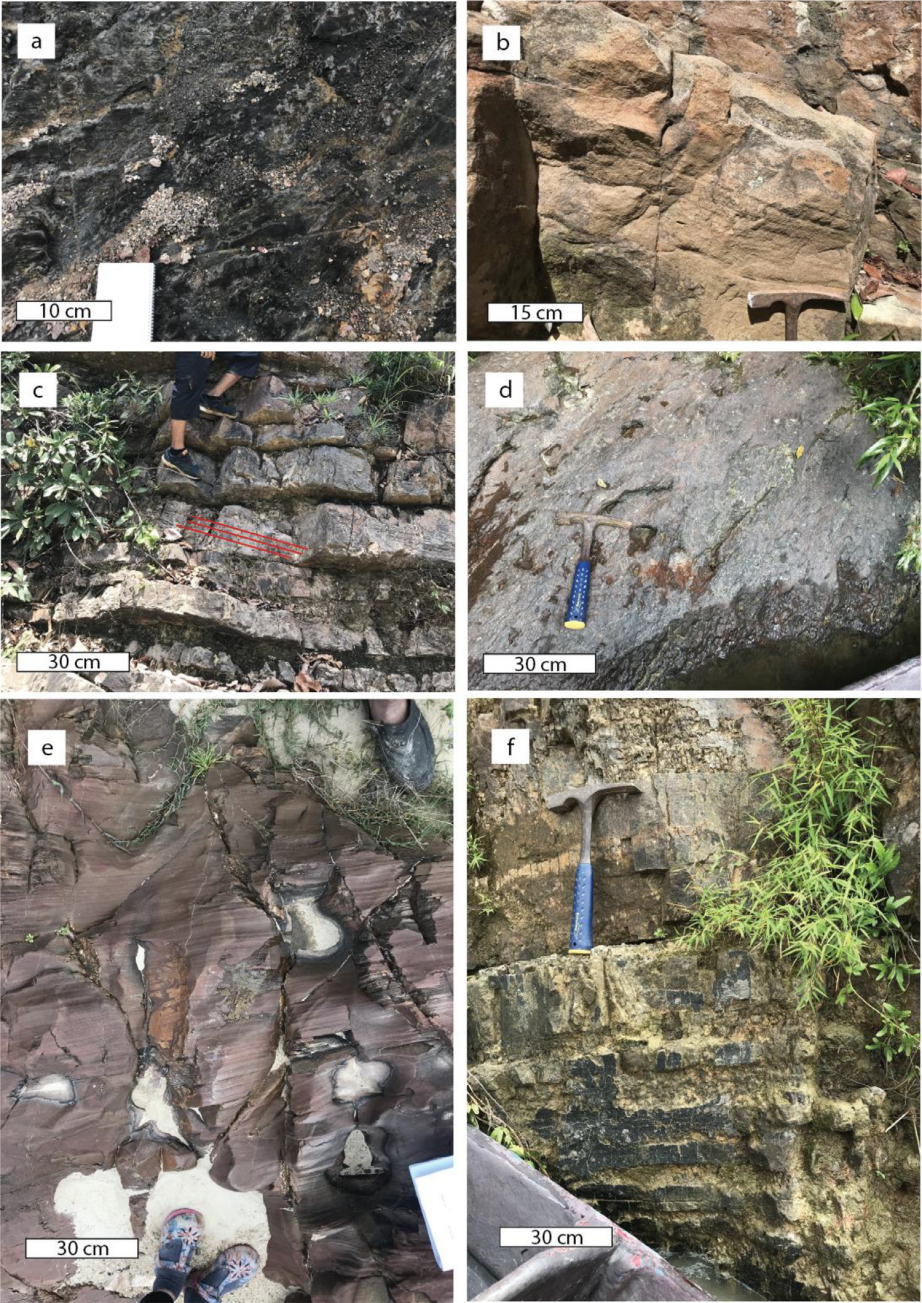
Compilation of geological structures in this study area. a) laminated mudstone (Fm); b) cross-bedded sandstone; c) planar cross-bedded sandstone (Sp); d) mudstone (Fm); e) parallel laminated sandstone (Sh); and, f) interbedded sandstone and mudstone (Fl).

## Limitations

One common limitation of sedimentary logging is that some beds may not preserve the structures or features present in the original sedimentary deposit. This could be due to various factors such as post-depositional changes, erosion, bioturbation, and diagenesis (chemical and physical changes during burial), some sedimentary structures may be altered or completely obliterated. These structureless beds are recorded as Structureless Sandstone Facies (Sm).

## Ethics Statement

This work did not involve studies with animals or human. The authors declare that they have no known competing financial interests or personal relationships which have or could be perceived to have influenced the work reported in this article.

## CRediT authorship contribution statement

**Wei Chung Khor:** Conceptualization, Methodology, Writing – review & editing, Funding acquisition. **Kamal Roslam Mohamed:** Writing – review & editing, Funding acquisition, Supervision. **Mohamed Shafeea Leman:** Funding acquisition, Supervision. **Che Aziz Ali:** Supervision.

## Data Availability

Kuala Tahan Sedimentary Logs and Data (Original data) (Mendeley Data)
